# The effect of antibiotics on post-adenotonsillectomy morbidity in Tanzanian children: study protocol for a randomized, double-blind, placebo-controlled trial

**DOI:** 10.1186/s13063-019-3830-5

**Published:** 2019-12-09

**Authors:** Denis R. Katundu, Peter S. Shija, Baltazari Nyombi, Hadija Semvua, Fieke K. Oussoren, Niels van Heerbeek

**Affiliations:** 1 Department of Otolaryngology, Kilimanjaro Christian Medical Centre, Kilimanjaro, Tanzania; 2 Department of Otolaryngology, Kilimanjaro Christian Medical University College, Kilimanjaro, Tanzania; 3 Department of Microbiology, Kilimanjaro Christian Medical University College, Moshi, Tanzania; 4 Department of Pharmacy, Kilimanjaro Christian Medical Centre, Moshi, Tanzania; 5 Department of Otolaryngology, Head and Neck Surgery, Radboudumc, Nijmegen, The Netherlands

**Keywords:** Adenotonsillectomy, Tonsillectomy, Postoperative morbidity, Amoxicillin, Randomized controlled trial, Antimicrobial resistance

## Abstract

**Background:**

Adenotonsillectomy is the most frequently performed operation in children worldwide. For decades, prophylactic antibiotics have been prescribed to limit postoperative complications. The effect of this antibiotic use has been refuted in a Cochrane Review. However, all reviewed studies were carried out in developed countries. In Tanzania, like many other developing countries with limited resources and a high burden of infectious diseases, postoperative antibiotic prescription is still very common to decrease the supposed higher postoperative morbidity. However, as a consequence of this widespread use of postoperative antibiotics, cross-resistance and risk of allergic side effects rise. Well-designed randomized controlled trials are needed to limit unnecessary antibiotic prescription and secondary antibiotic resistance.

**Aim:**

The aim of this study is to analyse the prophylactic role of postoperative antibiotics on morbidity following adenotonsillectomy in children in Tanzania.

**Design:**

The double-blinded, randomized, placebo-controlled trial was set in northern Tanzania. Participating centres are the Department of Otolaryngology at Kilimanjaro Christian Medical Centre in Moshi and the Department of Paediatric Surgery at the Arusha Lutheran Medical Centre in Arusha.

**Methods:**

Around 270 children aged 2–14 years, all scheduled for elective (adeno)tonsillectomy, will be included and assigned to receive either a standard regimen of 5 days of antibiotic prophylaxis or placebo after surgery. The primary outcomes are postoperative haemorrhage, fever and pain. Secondary outcomes are the time until normal diet is resumed, the time until normal activities are resumed and the occurrence of adverse events and microbial recolonization of the tonsillar beds.

**Discussion:**

This study will enhance an increase of proper antimicrobial prescription in Tanzanian institutions as well as other resource-limited countries where prescription of antibiotics is still very common. In addition, it might augment current knowledge about surface and core tonsillar micro-organisms and sensitivity patterns.

**Trial registration:**

Pan African Clinical Trials Registry, PACTR201905466349317. Retrospectively registered on 15 May 2019. https://pactr.samrc.ac.za/TrialDisplay.aspx?TrialID=8119

## Background

Adenotonsillectomy (ATE) is one of the most frequently performed operations in children worldwide and the most frequently performed otolaryngological operation in children. Indications for adenotonsillectomy are, amongst others, adenotonsillar hypertrophy, recurrent adenotonsillitis and obstructive sleep apnoea syndrome [1, 2]. This also applies to Tanzania, where both paediatric sleep apnoea as well as upper respiratory tract infections due to chronic adenotonsillar hypertrophy are very common problems. At Kilimanjaro Christian Medical Center (KCMC) alone, a large referral hospital in northern Tanzania, over 1000 adenotonsillectomies are performed yearly.

Different surgical techniques can be used to remove the tonsils and adenoid but, regardless of the surgical technique, the pharyngeal wall embedding the tonsillar fossa is left open for secondary wound healing at the end of the procedure. Subsequently, the wound bed is contaminated by commensal flora present in the oropharyngeal mucosa. It has been argued that, because of this, people are predisposed to inflammatory response and infection. This contributes to postoperative morbidity such as pain and the consequent need for analgesics, postoperative haemorrhage and the inability to resume a normal diet. For this reason, several studies have recommended the prophylactic use of antibiotics to reduce morbidity [2–5].

However, in a Cochrane Review published in 2012, involving 10 trials with a pooled population of 1035 patients, the use of postoperative antibiotics did not show a clinically relevant effect on the haemorrhage rates, the time to resume a normal diet, pain and consequent need for analgesics [5]. Little evidence suggested that prophylactic use of postoperative antibiotics reduced fever. With the existing set of evidence, Dhiwakar et al. advocated against the routine prescription of antibiotics to patients undergoing adenotonsillectomy. However, they did highlight the need for further trials including subgroups of patients who might be in need of selective administration [5]. None of the studies reviewed in this Cochrane Review had been conducted in resource-limited settings. Despite the lack of high-level evidence for postoperative antibiotics, in Tanzania all children receive postoperative antibiotics after adenotonsillectomy based on the assumption that there is a higher risk of postoperative morbidity due to limited resources and a higher burden of infectious diseases. On the other hand, the widespread use of (postoperative) antibiotics is not without risks. There is the individual risk of gastrointestinal and allergic side effects, ranging from vomiting, diarrhoea and rash to severe anaphylaxis. Also, there is the risk of antimicrobial resistance. While global concern for antimicrobial resistances rises, there is still a significant gap of knowledge on this topic in resource-limited settings, like Tanzania. In developing countries, amoxicillin is one of the most misused and wrongly prescribed drugs. The majority of young children suffering from acute respiratory infection symptoms are treated inappropriately with this antibiotic. Cross-sectional research from 2018, performed in Moshi, Tanzania, found that 92.3% of retailers dispensed antibiotics without prescriptions [6]. This practice disadvantages most inhabitants of these areas in particular. Cross-resistance and cost in obtaining superior antimicrobial agents become a big challenge.

Summarizing the aforementioned, the widespread use of postoperative antibiotics after (adeno)tonsillectomy in children in Tanzania may have more negative effects than benefits, if there are any benefits at all. To limit unnecessary antibiotic prescription and secondary antibiotic resistance, well-designed randomized controlled trials are needed. This randomized double-blind placebo-controlled trial studies the prophylactic effect of antibiotics on morbidity after adenotonsillectomy in children in Tanzania.

## Method

### Design

This study is a two-centre, double-blind, randomized controlled trial. Patients will be randomly allocated to receive either 50 mg/kg body weight of placebo or amoxicillin 5 days after surgery as the standard prophylaxis regime.

### Study setting

Participating centres are the Department of Otolaryngology at Kilimanjaro Christian Medical Centre (KCMC) in Moshi and the Department of Paediatric Surgery at the Arusha Lutheran Medical Centre (ALMC) in Arusha, the only settings with established ear, nose and throat (ENT) services in northern Tanzania.

### Study population

All children aged 2–14 years who will undergo an elective (adeno)tonsillectomy will be approached. Elective surgery is indicated in the case of recurrent chronic tonsillitis, as defined by five or more episodes of tonsillitis yearly for at least 2 consecutive years, or in the case of obstructive sleep apnoea due to adenotonsillar hypertrophy with inadequate response to pharmacotherapy. Inclusion will occur after written informed consent. Inclusion of patients started in January 2019.

Patients will be excluded from participation if they meet any of the following criteria:

- The patient is scheduled for unilateral tonsillectomy, tonsillar biopsy or tonsillectomy for a known carcinoma.
- The (adeno)tonsillectomy is combined with any other type of surgery.
- The patient has an acute tonsillar infection or peritonsillar abscess at the time of surgery.
- The patient is known to have a cardiac co-morbidity, with or without current treatment or follow-up by a cardiologist.
- The patient has a condition which results in weakness or failure of the host defence mechanism, like leukaemia, acquired immune deficiency syndrome or current use of immunosuppressive medication.
- The patient has a syndromal disorder or a craniofacial malformation.
- The patient has a reported allergy to any antibiotic, ibuprofen or paracetamol.
- The patient was treated with antibiotics during or less than 1 week prior to surgery.
- Patients on any other concomitant medications at the point of enrolment.

### Study objective

The study objective is to compare the postoperative morbidity following elective (adeno)tonsillectomy in children treated with postoperative amoxicillin or a placebo in northern Tanzania. We hypothesize that placebo is non-inferior to amoxicillin in preventing postoperative morbidities following (adeno)tonsillectomy in children in northern Tanzania.

### Primary outcomes

The primary outcome is post-(adeno)tonsillectomy morbidity, but morbidity cannot be measured as a single parameter. Postoperative haemorrhage and infection are the most important postoperative complications contributing to post-(adeno)tonsillectomy morbidity. Post-(adeno)tonsillectomy infection is difficult to define since the tonsillar beds are left to heal by secondary intention after surgery in a bacterial, mucosal environment. Typical symptoms of surgical site infection are therefore absent. Clinically worsening pain and raised temperature are thus considered features of infection. For this reason, postoperative haemorrhage, raised temperature (fever) and pain are the primary outcomes in this research.

Postoperative haemorrhage is defined using two parameters: major if warranting re-admission, blood transfusion or return to theatre for haemostasis; and minor if any recorded postoperative blood loss (i.e. spitting of blood saliva 24 h post operation). Fever is defined as temperature greater than 38 °C on 2 consecutive postoperative days or greater than 39 °C on any postoperative day, and postoperative pain is rated using the Wong-Baker FACES® Pain Rating Score.

Temperature will be taken daily by the research nurse while admitted and thereafter by the parent/caretaker with an axillary thermometer until the 7th postoperative day.

The Wong-Baker FACES® Pain Rating Scale will be used to assess children’s pain perception postoperatively. Written permission has been granted from Wong-Baker FACES Foundation for using both the English (while in hospital) and Swahili (while at home) Wong-Baker FACES® Pain Rating Scale in this study. The consequent need for analgesics will be documented by the prescribing physician.

### Secondary outcomes

The secondary outcomes include the time until normal diet is resumed, time until normal activities are resumed and occurrence of adverse events (rash, vomiting, diarrhoea and anaphylaxis) and microbial recolonization of the tonsillar niche.

### Study sample

Postoperative bleeding, fever and pain were used as primary outcomes. We calculated power for all primary outcomes and only took the outcome with the largest number of participants. Based on the latest Cochrane Review, titled “Antibiotics to reduce post-tonsillectomy morbidity” [5], 2.5% of all patients who undergo an elective tonsillectomy have a significant postoperative haemorrhage. With this haemorrhage rate, a significance level (α) of 0.05, a power (π) of 0.80 and a non-inferiority margin of 5%, we calculated the number of patients in our intervention group. The group treated with antibiotics should include 121 patients, giving a total of 242 patients necessary for analysis. To allow for some loss to follow-up, the aim is to include 270 children.

### Randomization

Baseline characteristics of the subjects will be compiled at informed consent. Subjects will be randomly divided into an intervention arm and a control arm on the day of admission, with the aid of a computer-based randomization module in a 1:1 ratio. Stratified randomization is accomplished using gender, age group (2–4 years, 5–8 years and 9–14 years), residence (rural or urban) and research centre (KCMC or ALMC) as strata. Randomization is performed by the KCMC’s research pharmacist who will then prepare the medication (either the antibiotics or placebo capsules or solution, depending on the age of the child). Both the placebo capsules as well as solution have the same taste and appearance as the amoxicillin capsules and solution. Labelled with only the study protocol number and patients’ details, the medication will be distributed by one of the research nurses. Thus, all health professionals involved in the trial process will be blinded.

### Surgery

All (adeno)tonsillectomies will be carried out under general anaesthesia with the use of orotracheal intubation. Surgery will be performed by all grades of doctors, from residents to specialists. The tonsils are removed using either routine dissection, Sluders technique or electrodissection [7]. Which technique is chosen depends on the surgeon’s experience and preference. Previous studies have shown that the technique does not affect the outcome or the postoperative morbidity [7, 8]. In all patients, the adenoid is removed using a Beckmann adenoid curette.

### Microbial isolates

During surgery, just before dissection of the tonsils, a swab (Copan ESwab; Copan Italia SPA, Brescia, Italy) will be taken from the tonsillar surface for culture and sensitivity. After the operation is completed, one dissected tonsil will be submerged in iodopovidone for around 30 s. Subsequently, the tonsil will be rinsed using sterilized normal saline and divided into two parts with a new sterile blade. Another swab (Copan ESwab; Copan Italia SPA) will be taken from the core of the tonsil.

For patients operated at KCMC, both swabs will be maintained at a temperature between 2 and 8 °C and dispatched within 30 min to the microbiology research laboratory. Samples from ALMC will be stored at a temperature between 2 and 8 °C and transported on the same day to the microbiology research laboratory. At the microbiology research laboratory, all swabs will be incubated at 37 °C for 24 h. After incubation is completed, subcultures from growing microorganisms will be prepared on agar plates. The isolated bacteria will then be Gram stained and microscopically investigated. Only pathogenic organisms will be worked up, no work-ups for normal flora will be done. Microbiological work-up will entirely adhere to the Clinical and Laboratory Standards Institute (CLSI) Performance Standards for Antimicrobial Susceptibility Testing. Antibiotic sensitivity testing will be done for all pathogenic, cultured microorganisms using the disc diffusion test [9]. Discs of the most common antibiotics will be used.

### Postoperative management

After surgery, all patients will be kept in the ward for at least one night. Based on their clinical condition, patients will be discharged. On the ward, patients will receive their labelled medication with either 50 mg/kg amoxicillin or placebo. Medication will be taken 8-hourly for 5 consecutive days. Parents and caretakers are given instructions about the importance of continuing the medication after discharge from the hospital.

All patients will be given paracetamol three times a day and ibuprofen twice a day based on their weight for 10 days. Any additional analgesics or other treatment will be started whenever necessary at the discretion of the treating physician.

### Follow-up

During discharge, a questionnaire will be provided to the persons legally responsible for the participating child. This will be filled out daily and will be presented to the investigator at follow-up visits. The questionnaire is translated, adopted and modified from the standardized Linden et al. [10] post-tonsillectomy follow-up questionnaire (Additional file 1).

**Fig. 1:**
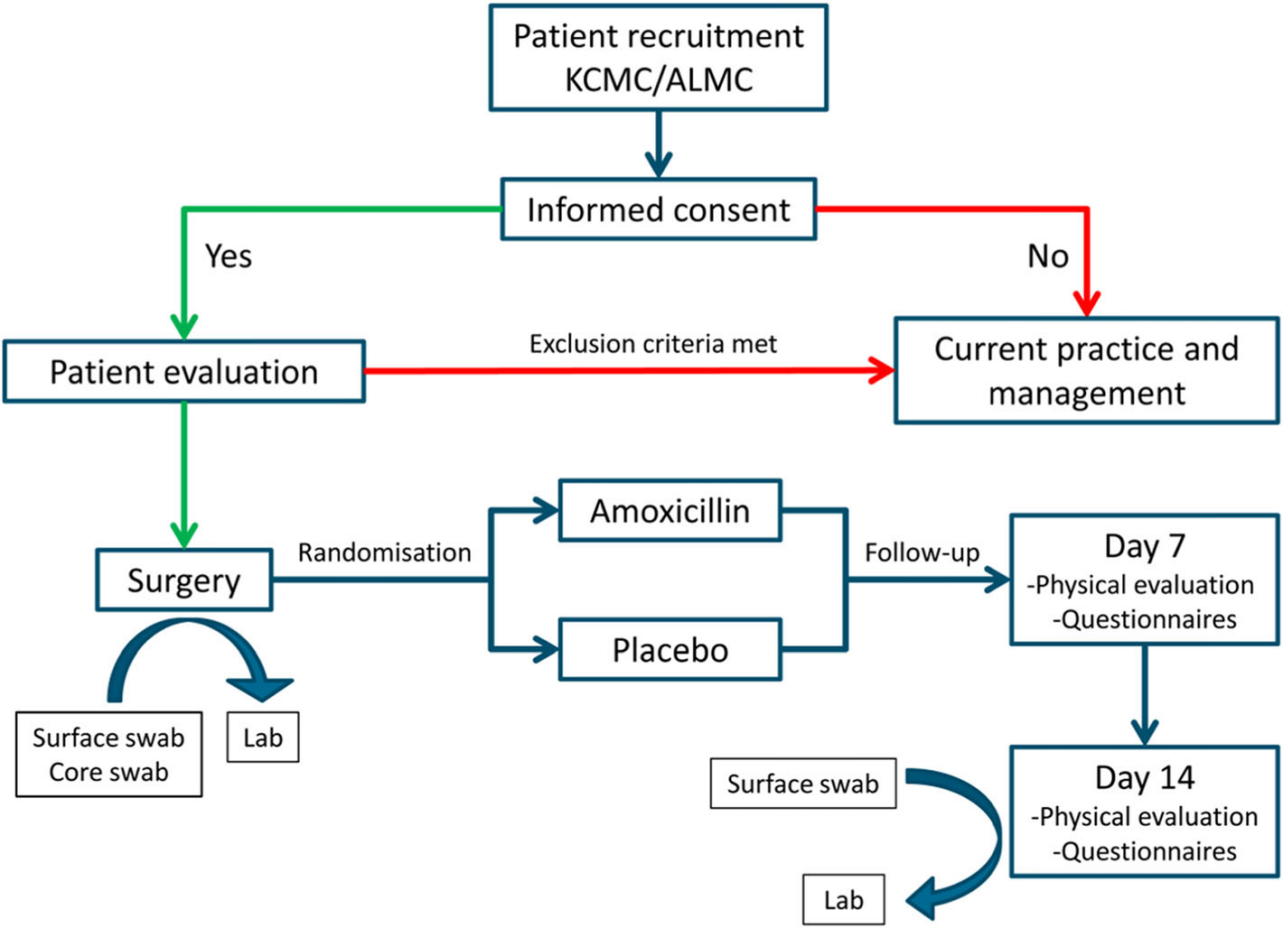
Flow chart of the trial process from allocation to follow-up. ALMC Arusha Lutheran Medical Centre, KCMC Kilimanjaro Christian Medical Centre

**Fig. 2:**
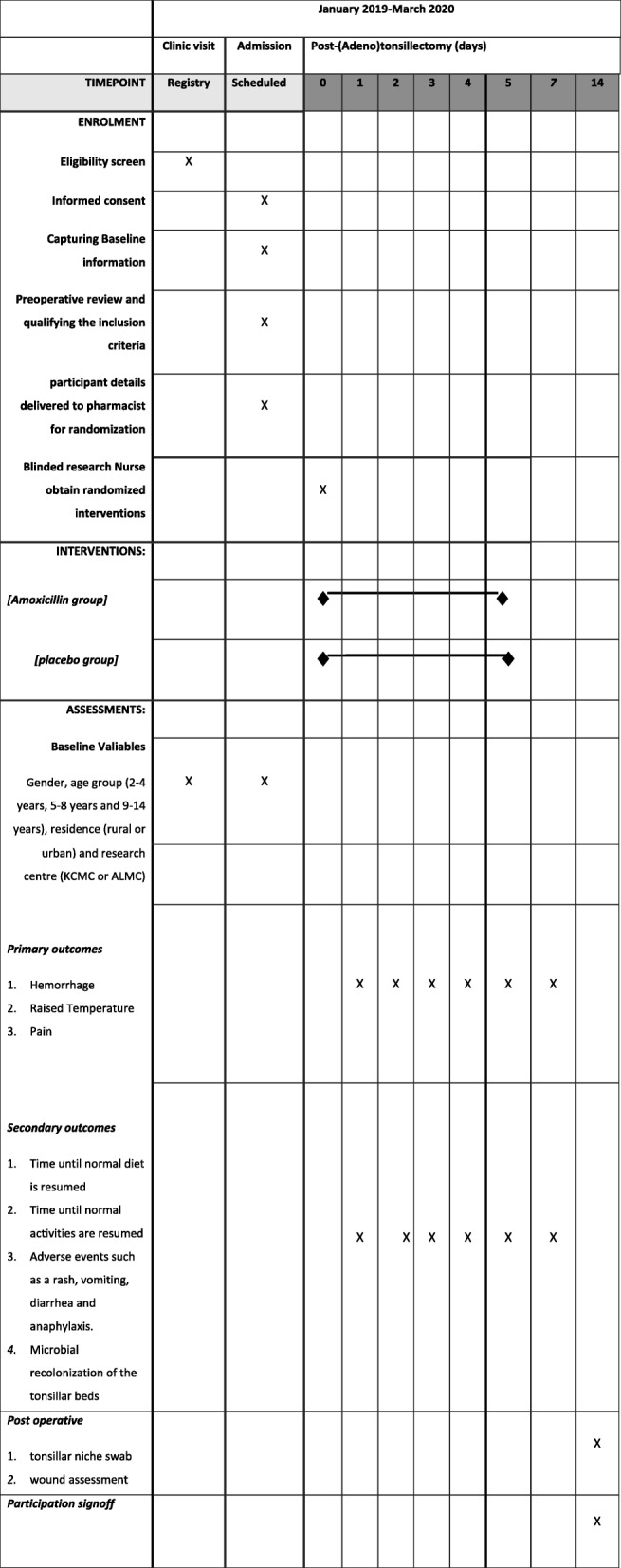
Standard Protocol Items: Recommendations for Interventional Trials (SPIRIT) figure of the trial schedule for enrolment, interventions and follow-up/assessments. ALMC Arusha Lutheran Medical Centre, KCMC Kilimanjaro Christian Medical Centre

On day 7, the wound bed and the general health status will be inspected by the study doctor at the Department of Otolaryngology at KCMC and ALMC respectively. On day 14, a third swab (Copan ESwab; Copan Italia SPA) of the surgical site will be taken and sent to the microbiology research laboratory for culture and sensitivity analysis, as already described. During both visits, detailed information will be obtained by the study doctor. The entire process from patient allocation to follow-up is displayed in Fig. 1 and well described in a SPIRIT (Standard Protocol Items: Recommendations for Interventional Trials) figure in Fig. 2.

### Statistical analysis

Continuous variables will be presented as means and standard deviation or median and interquartile range (IQR) if not normally distributed. Categorical data will be presented as a number with percentage. All analysis will be done according to an intention-to-treat protocol.

Differences between the observed risks of events (for primary outcomes of bleeding and fever) between the placebo and antibiotics groups will be calculated along with their 95% confidence intervals. Absolute risks will also be presented as it is important to be aware of the underlying risk of events. Differences in pain score between the two treatment arms will be calculated as mean differences with 95% CIs, using Student’s *t* tests. Secondary outcomes will be analysed using chi-squared tests for categorical data (return to normal activities and microbial recolonization) or Wilcoxon rank-sum tests (days to return to normal diet) for continuous data. *P* < 0.05 will be considered statistically significant. All data will be coded to maximize confidentiality. Collected data will be entered into a database and analysed using IBM SPSS Statistics version 24.

### Data and safety monitoring

Data and safety monitoring will be done by the independent data and safety monitoring committee of Kilimanjaro Christian Research Institute (KCRI). They will perform interim analyses every 6 months as long as patient inclusion and data collection are ongoing.

### Ethics approval and consent to participate

This clinical trial has been approved by the Kilimanjaro Christian Medical College Research Ethics and Review Committee (CRERC), the Tanzanian Food and Drug Authority (TFDA) and the Tanzanian National Institute for Medical Research (NIMR). Prior to randomization, written informed consent will be obtained from the parents/caretakers of each participant.

## Discussion

### Study strengths

Our study aims to evaluate the effect of antibiotic prophylaxis on postoperative morbidity following (adeno)tonsillectomy in Tanzania. This research could add to the generalization and increase in proper antimicrobial prescription in institutions in Tanzania as well as in the whole of Africa. Furthermore, it might enhance current knowledge about surface and core tonsillar micro-organisms.

A young age, poor nutritional status and either very low or very high body weight are likely contributing factors to worse outcomes after (adeno)tonsillectomy. Moreover, in a developing country like Tanzania, there is significant inequality in living conditions and overall health status between the general public and the upper class. Children raised in a low social economical environment are prone to higher morbidity rates regardless of the postoperative management.

Through the use of randomization in this study design, we aim at limiting these confounders. Furthermore, we use double blinding to prevent information bias by both subjects and researchers.

### Study limitations

Children coming from distant areas surrounding the hospital might not return for follow-ups and therefore the loss to follow-up rate might be higher than estimated. This in turn could result in inadequate sample sizes. We will try to reduce the amount of subjects lost to follow-up by implementing reimbursement of travel expenses.

Different surgical techniques will be used in performing (adeno)tonsillectomy. This can be routine dissection, Sluder technique or electrodissection, according to the preference and custom of the surgeon performing the operation. As a consequence of this, the use of coagulation may differ within subjects. This, in turn, could result in differences in prevalence of pain and infection postoperatively. Still, up to now there are insufficient data to prove that one method of tonsillectomy is superior to the others [8].

In Tanzania, it is a custom that relatives oversee patients’ medication supply and intake. Although all patients are prescribed the same amount of analgesics, it is not regular practice that nurses supervise the medication intake. This could influence the patients’ perception of pain. Moreover, flushing of the wound bed with water postoperatively might decrease the infection risk following (adeno)tonsillectomy. This, however, is a management factor which is difficult to standardize and could therefore influence the outcomes.

### Trial status

This is protocol version 9 October 2019, v12_2019. At the time of manuscript submission, the researchers have started recruiting and including patients. The anticipated date of last recruitment is 27 February 2020.

## Supplementary information

**Additional file 1.** SPIRIT 2013 Checklist: Recommended items to address in a clinical trial protocol and related documents.

## Abbreviations

ALMC: Arusha Lutheran Medical Centre
CRERC: Kilimanjaro Christian Medical College Research Ethics and Review Committee
ENT: Ear, nose and throat
IQR: Interquartile range
KCMC: Kilimanjaro Christian Medical Centre
NIMR: Tanzanian National Institute for Medical Research
PACTR: Pan African Clinical Trials Registry
SPIRIT: Standard Protocol Items: Recommendations for Interventional Trials
TFDA: Tanzanian Food and Drug Authority
