# Supplementary material for: The effect of antibiotics on post-adenotonsillectomy morbidity in Tanzanian children: study protocol for a randomized, double-blind, placebo-controlled trial
Source: Trials. 2019 Dec 9;20:683. doi: 10.1186/s13063-019-3830-5 (PMC6902588; doi:10.1186/s13063-019-3830-5)
Supplement: Supplementary file 1 — Additional file 1. SPIRIT 2013 Checklist: Recommended items to address in a clinical trial protocol and related documents. [file 13063_2019_3830_MOESM1_ESM.docx]

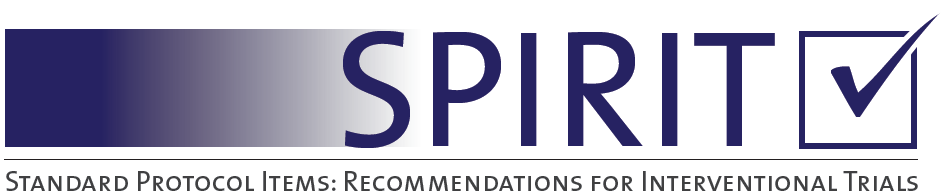


SPIRIT 2013 Checklist: Recommended items to address in a clinical trial protocol and related documents*

| Section/item | ItemNo | Description |
| --- | --- | --- |
| **Administrative information** | | |
| Title | 1 | The Effect of Antibiotics On Post-Adenotonsillectomy Morbidity In  Tanzanian Children: Study Protocol for A Randomized Double-blind  Placebo Controlled Trial. Page 01 |
| Trial registration | 2a | Trial registered to Pan African Clinical Trials Registry (PACTR) with Trial registration number PACTR201905466349317, registered Retrospectively on 15 May 2019. Page 03 |
|  | 2b | <https://pactr.samrc.ac.za/TrialDisplay.aspx?TrialID=8119> Page 03 |
| Protocol version | 3 | 9 October 2019, v12_2019. Page 11 |
| Funding | 4 | Radboud University Medical Centre Revolving Research Fund, a scholarship program for PhD candidates from low-middle income countries. Covering whole research budget. Page 12  Secondary Sponsors are Tanzania Ministry of Health Community  Development Gender Elderly and Children (funding postgraduate research activities) and Training Health Researchers into Vocational  Excellence THRiVE (covering publication related costs). Page 12 |
| Roles and responsibilities | 5a | Denis Katundu and Niels van Heerbeek designed the study and drafted the manuscript. Denis Katundu performs inclusion and follow-up of all patients and participates in surgical procedures. Peter Shija is local supervisor and participates in surgical procedures. Baltazar Nyombi and Hadija Semvua are responsible for microbiology and trial pharmacist respectively. Peter Shija, Baltazar Nyombi, Hadija Semvua and Fieke Oussoren coauthored the manuscript. Page 12 |
|  | 5b | Radboud University Medical Centre Revolving Research Fund  P.O. Box 9101, 6500 HB Nijmegen  The Netherlands  lnternal postal code 630  Geert Grooteplein Zuid 10  Radboudumc hoofdingang, route 630  T +31 24 818 69 11  Attention: Prof. Paul A.B.M. Smits, MD, PhD |
|  | 5c | Radboud University Medical Centre Revolving Research Fund as Primary Funder is committed in financing whole research budget. Tanzania Ministry of Health Community Development Gender Elderly and Children (MoHCDEC) together with Training Health Researchers into Vocational Excellence (THRiVE) as Secondary Sponsors are responsible for funding postgraduate research activities including local related fees and publication related costs respectively. All these monetary bodies are purely not associated and engaged with study design; collection, management, analysis, and interpretation of data; writing of the report; and the decision to submit the report for publication. Page 12 |
|  | 5d | Not Applicable.  As According to study design and study objectives the National Health Research Ethics Committee under National Institute for Medical Research |
| Introduction |  |  |
| Background and rationale | 6a | Adenotonsillectomy (ATE) is one of the most frequently performed operations in children worldwide and the most frequently performed otolaryngological operation in children. Indications for adenotonsillectomy are, amongst others, adenotonsillar hypertrophy, recurrent adenotonsillitis and obstructive sleep apnea syndrome[1, 2]. This also applies to Tanzania, where both pediatric sleep apnea as well as upper respiratory tract infections due to chronic adenotonsillar hypertrophy are very common problems. At Kilimanjaro Christian Medical Center (KCMC) alone, a large referral hospital in Northern Tanzania, about 1000 adenotonsillectomies performed yearly.  Different surgical techniques can be used to remove the tonsils and adenoid but regardless of the surgical technique, the pharyngeal wall embedding the tonsillar fossa is left open for secondary wound healing intention at the end of the procedure. Subsequently the wound bed is contaminated by commensal flora present in the oropharyngeal mucosa. It has been argued that, because of this, people are predisposed to inflammatory response and infection. This contributes to postoperative morbidity such as pain and the consequent need for analgesics, postoperative haemorrhage and the inability to resume normal diet. For this reason, several studies have recommended the prophylactic use of antibiotics to reduce the morbidity [2-5].  However, in a Cochrane review published in 2012, involving 10 trials with a pooled population of 1035 patients, the use of postoperative antibiotics did not show a clinically relevant effect on the haemorrhage rates, the time to resume a normal diet, pain and consequent need for analgesics[5]. Little evidence suggested that prophylactic use of postoperative antibiotics reduced fever. With the existing set of evidence, Dhiwakar and colleagues advocated against the routine prescription of antibiotics to patients undergoing adenotonsillectomy. However, they did highlight the need for further trials including subgroups of patients who might be in need of selective administration[5]. None of the studies reviewed in this Cochrane review had been conducted in resource-limited settings. Despite the lack of high level evidence for postoperative antibiotics, in Tanzania all children receive postoperative antibiotics after adenotonsillectomy based on the assumption that there is a higher risk of postoperative morbidity due to limited resources and a higher burden of infectious diseases. On the other hand, the widespread use of (postoperative) antibiotics is not without risks. There is the individual risk of gastrointestinal and allergic side effects, ranging from vomiting, diarrhoea and rash to severe anaphylaxis. And there is the risk of antimicrobial resistance. While global concern for antimicrobial resistances rises, there is still a significant gap of knowledge on this topic in resource-limited settings, like Tanzania. In developing countries, amoxicillin is one of the most misused and wrongly prescribed drugs. The majority of young children suffering from acute respiratory infection symptoms is treated inappropriately with this antibiotic. A cross-sectional research from 2018, performed in Moshi Tanzania, found that 92.3% of retailers dispensed antibiotics without prescriptions [6]. This practice disadvantages most inhabitants of these areas in particular. Cross-resistance and cost in obtaining superior antimicrobial agents become a big challenge.  Summarizing the above, the widespread use of postoperative antibiotics after (adeno)tonsillectomy in children in Tanzania may have more negative effects than benefits, if there are any benefits at all. To limit unnecessary antibiotic prescription and secondary antibiotic resistance, well-designed randomized controlled trials are needed. This randomized double-blind placebo-controlled trial studies the prophylactic effect of antibiotics on morbidity after adenotonsillectomy in children in Tanzania.  Page 04 and 05 |
|  | 6b | Amoxicillin which has been used as a post (adeno)tonsillectomy standard prophylaxis antibiotic drug will be compared with a placebo made available from the pharmacy which doesn’t have any therapeutic ingredient.  Page 04 and 05 |
| Objectives | 7 | Broadly we are comparing the postoperative morbidity following elective (adeno)tonsillectomy in children treated with postoperative amoxicillin or a placebo in Northern Tanzania. Page 06  Specific Objectives   1. To compare co-morbidities of post tonsillectomy between the two arms. 2. To describe surface and core tonsillar microbial isolates among children undergoing tonsillectomy. 3. To describe susceptibility pattern of surface and core tonsillar microbial isolates among children undergoing tonsillectomy. 4. To compare occurrence of halitosis, fever and pain in first seven days post surgery between amoxicillin prophylaxis and placebo arm. 5. To compare the occurrence of amoxicillin sensitive microorganisms in the tonsillar fossa 07 days postoperative between two groups.   Page 06  We hypothesize that placebo is noninferior to amoxicillin in preventing postoperative morbidities following a (adeno)tonsillectomy in children in northern Tanzania.  Page 06 |
| Trial design | 8 | Two centre, Factorial, noninferiority, double-blind, randomized placebo controlled trial. Page 05 |
| Methods: Participants, interventions, and outcomes | | |
| Study setting | 9 | Department of Ear, Nose and Throat of Kilimanjaro Christian medical Centre in Kilimanjaro and Department of Pediatrics Surgery of Arusha Lutheran Medical Centre in Arusha, Consultant and teaching Hospitals in Northern Tanzania. Page 05 |
| Eligibility criteria | 10 | All Children from 2 to 14 years of age who will undergo an elective (adeno)tonsillectomy. Page 05  All surgeries will be carried out under general anaesthesia with the use of orotracheal intubation. Surgeries being performed by all grades of surgeons from registrars, residents and consultants. Page 08 |
| Interventions | 11a | Children electively admitted for tonsillectomy will be randomly divided using a computer software into two groups on the day of admission: First group consists of children who received amoxicillin 50mg/kg body weight eight hourly in the suspension or tablets form for 5 days starting from the day of surgery, and second group consists of patients who will receive placebo. Page 05  According to randomization, patients’ details will be submitted to the appointed research pharmacist one day prior to surgery for procurement and preparation of antibiotic (placebo or active medication), medications will be then available for collection by the ENT nurse next day. Page 07 |
|  | 11b | All patients who develops allocated interventions side effects such for as penicillin sensitivity, Immediate post operative complication such as bleeding and requires to return to operating room or further intervention which necessitate change of allocated interventions, will be withdrawn from the study and local routine protocol will be continued.  Page 06 |
|  | 11c | While in the wards medications of allocated interventions will be labeled for each patient and nurses will dispense routinely as prescribed. As scheduled parents or persons legally responsible for the patients will be given medications and the instruction on administration and timing will given by a blinded research nurse. Concomitantly all patients in the trial will receive acetaminophen and ibuprofen per kilogram body weight for 10 days as per local post surgery pain management protocol. Full time Research dedicated cellphone number is given to all participants in case of any concern.  Page 07 |
|  | 11d | Printed post adeno(tonsillectomy) dietary and general care hand-out is given to all participants which explains all things allowed during the first 14 days post operative. Any concern during the followup period is communicated to the principle investigator through a all time available cellphone.  Page 06 |
| Outcomes | 12 | The primary outcome is post-(adeno)tonsillectomy morbidity, however morbidity cannot be measured as a single parameter. Postoperative haemorrhage and infection are the most important postoperative complications contributing to post-(adeno)tonsillectomy morbidity. Post-(adeno)tonsillectomy infection is difficult to define since the tonsillar beds are left to heal by secondary intention after surgery in a bacterial, mucosal environment. Typical symptoms of surgical site infection are therefore absent. Clinically worsening pain and raised temperature are thus considered features of infection. For this reason, postoperative haemorrhage, raised temperature (fever) and pain are the primary outcomes in this research.  Postoperative haemorrhage as defined using two parameters (major as if warranting re‐admission, blood transfusion or return to theatre for haemostasis and minor as any recorded postoperative blood loss i.e. Spitting of blood saliva 24 hours post-operative). Fever (temperature greater than 38°C on 2 consecutive post-operative days or greater than 39°C on any postoperative day) and Post-operative pain (Wong-Baker FACES® Pain Rating Score).  Temperature will be taken daily by the research nurse while admitted and thereafter by the parent/caretaker with an axillary thermometer until the seventh postoperative day.  The Wong-Baker FACES® Pain Rating Scale will be used to assess children's pain perception postoperatively. Written permission has been granted from Wong-Baker FACES Foundation for using both the English (while in hospital) and Swahili (while at home) Wong-Baker FACES® Pain Rating Scale in this study. The consequent need for analgesics will be documented by the prescribing physician.  Secondary outcomes  These includes time until normal diet is resumed, time until normal activities are resumed and adverse events (rash, vomiting, diarrhea and anaphylaxis) and microbial recolonization of the tonsillar niche.  Page 06 |
| Participant timeline | 13 | Time schedule of enrolment, interventions and followup of participants well stipulated (see Figure 1)  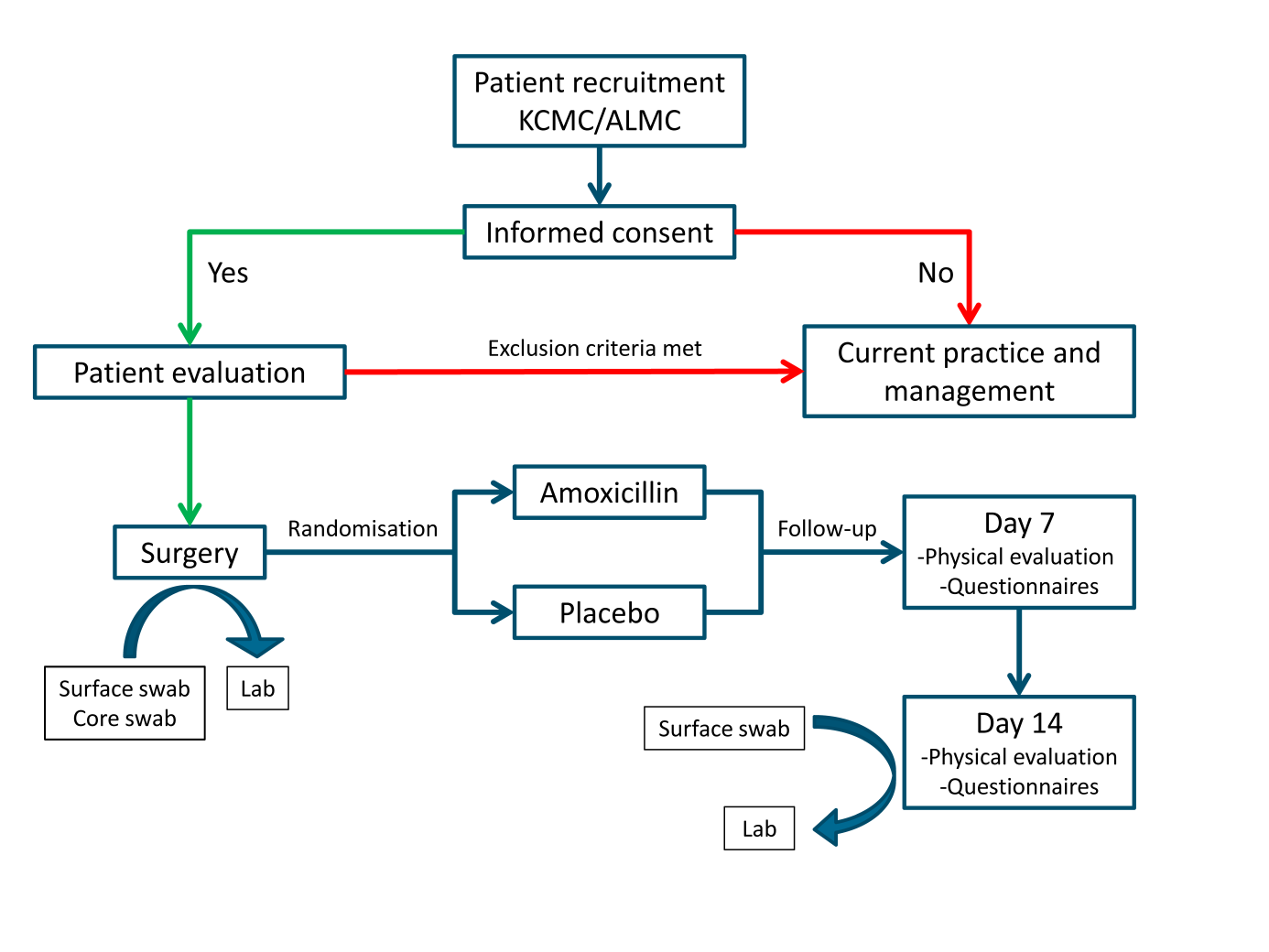Figure 1: Flow chart of the trial process from allocation to follow-up.  Page 07 and 08 |
| Sample size | 14 | Basing on Post-operative bleeding, infection (fever) and pain as primary outcomes. We calculated power for all primary outcomes and only took the outcome with largest number of participants. Based on the latest Cochrane review, titled “Antibiotics to reduce post-tonsillectomy morbidity”, 2,5% of all patients who undergo an elective tonsillectomy have a significant postoperative haemorrhage. With this haemorrhage rate, a significance level (α) of 0.05, a power(π) of 0.80, and a non-inferiority margin of 5%, we calculated the number of patients in our intervention group. The group treated with antibiotics should include 121 patients, giving a total of 242 patients necessary for analysis. To allow for some loss to follow-up, the aim is to include 270 children.  Page 07 |
| Recruitment | 15 | Thorough insisting about the importance of finishing the study follow-up at the time of enrolment. Including patient who don’t travel more than around 80km from any of the two centres. Time to time visit reminding texts to participants’ parent/caretaker. Reimbursement of bus fair and hospital consultation fee to the participant.  Page 08 |

| **Methods: Assignment of interventions (for controlled trials)** | | |
| --- | --- | --- |
| Allocation: |  |  |
| Sequence generation | 16a | Block randomisation with at random variable block size of 6 and 8 patients. Baseline characteristics of the subjects will be compiled at informed consent. Subjects will be randomly divided into an intervention and a control arm on the day of admission, with the aid of a computer-based randomization module in a 1:1 ratio. Stratified randomization is accomplished using gender, age group (2-4 years, 5-8 years and 9-14 years), residence (rural or urban) and research centre (KCMC or ALMC) as strata.  Page 07 |
| Allocation concealment mechanism | 16b | Central randomisation by phone/fax.  Page 07 |
| Implementation | 16c | Basing on the department elective surgery registry book, parents/caretakers of all children who are electively scheduled for (adeno)tonsillectomy will be approached though their registered mobile phone numbers by the research investigator and the research will be explained to them and they will be asked for an opportunity to participate observing their autonomy and right to deserve a scheduled treatment regardless their informed choice, if they accept, they will be further consented and enrolled into the study by the principal investigator on the day of admission.  On the day of admission, patients Baseline characteristics will be sent to randomizing pharmacist. And he will be responsible in allocated participants to either amoxicillin or placebo group. Randomization number will then be returned to the principal investigator for documentation.  Page 08 |
| Blinding (masking) | 17a | As all the randomization will be done by pharmacist, This will give no opportunity to the whole research team and parent/caretaker to know what arm does the participant belong up to the point of analysis as data analysis will also be done by an independently biostatistician.  Page 07 |
|  | 17b | If undesirable outcomes such for example anaphylactic events of hypersensitivity or if features of sepsis are encountered. Study supervisor and ENT resident doctor on call for that specific time will have an opportunity to access a participant’s allocated intervention group for proper management. Keeping all the medical records in track and registered for study as well.  Page 11 |

| **Methods: Data collection, management, and analysis** | | |
| --- | --- | --- |
| Data collection methods | 18a | Case report forms will be used to register and document patients information and persons legally responsible for the patients will be given a questionnaire to be answered daily for the progress of patient for seven days and presented each scheduled follow-up, follow-ups logs and patients progressive file notes will be filled on each visit and documented in research database.  During discharge, after clear explanation on how to handle it, a self-administered translated, adopted and modified Linden et al post tonsillectomy follow-up questionnaire will be provided to persons legally responsible for the participating child. It will be answered and filled on daily basis, presented to the investigator on every visit.  Wong-Baker FACES® Pain Rating Scale will be used in assessing postoperative children's pain perception. Written permission has been granted from Wong-Baker FACES Foundation for using Wong-Baker FACES® Pain Rating Scale in this study.  Daily tonsillar surface and core cultures results will be obtained from research laboratory, entered in both paper-based logs and study database.  Page 06, 07 and 09 |
|  | 18b | Continuous insisting about the importance of finishing the study follow-up at the time of enrolment. Including participant who don’t travel more than around 80km from any of the two centres. Time to time visit reminding texts to participants’ parent/caretaker. Reimbursement of bus fair and hospital consultation fee to the participant.  Double checking home-based questionnaires during hospital visit when they are returned and also exclude patients who deviate from intervention protocols. Intention to treat Analysis of lost to follow-up.  Page 06, 07,08 and 09. |
| Data management | 19 | Collected data will be entered into respective patient research paper based folder then entered to digital database in licensed IBM^®^ SPSS^®^ Statistics version 24 for cleaning and analysis. Password protected Backup will be created time to time in an external hard drive.  All research data will stored under lock and under the custody of the principal investigator and supervisor (s) to prevent any illicit access to the data. Use of coded data will be ensured to ensure maximum confidentiality. At the end of the study, raw data will be stored in one soft copy storage device and folders will be locked in a special cabin at ENT department doctors’ office for a specific period.  Page 09 and 10 |
| Statistical methods | 20a | Continuous variables will be presented as means and standard deviation or median and interquartile range (IQR) if not normally distributed. Categorical data will be presented as a number with percentage.  Differences between the observed risks of events (for primary outcomes bleeding and fever) between placebo and antibiotics group will be calculated along with their 95% confidence intervals. Absolute risks will also be presented as it is important to be aware of the underlying risk of events. Differences in pain score between the two treatment arms will be calculated as mean differences with 95% CIs, using Student’s tests. Secondary outcomes will be analysed using Chi squared tests for categorical data (return to normal activities and microbial recolonization) or Wilcoxon rank sum tests (days to return to normal diet) for continuous data. P-values < 0.05 will be considered statistically significant. All data will be coded to maximize confidentiality. Collected data will be entered into a database and analysed using IBM® SPSS® Statistics version 24. |
|  | 20b | All analysis will be done according to an intention-to-treat protocol. |
|  | 20c | Intention to treat analysis will employed to take charge of lost to followup.  Page 09 and 10 |
| **Methods: Monitoring** | | |
| Data monitoring | 21a | Data and safety monitoring will be done by the independent data and safety monitoring committee of Kilimanjaro Christian Research Institute (KCRI). |
|  | 21b | They will perform interim analyses every six months as long as patient inclusion and data collection is ongoing. The study will be stopped when interim analyses show that using placebo has a postoperative 20% more major bleeding in the placebo group than in the antibiotic group, the major bleeding (significant blood loss warranting re-admission, blood transfusion or return to theater for haemostasis). Again 20% more often fever in the placebo group. As Pain is indeed considered subjective and is normal postoperatively, hence no stopping rule. |
| Harms | 22 | Any patients who reported and confirmed to develop any undesirable outcome form the study drug such for example penicillin hypersensitivity, the medical care will be taken care by the research funds until recovered (this is as well explained during enrolment). |
| Auditing | 23 | Study auditing will be done by the Kilimanjaro Christian Medical College Research Ethics and Review Committee (CRERC) at any point during the study period without notifying the investigators.  Page 10 |
| Ethics and dissemination | | |
| Research ethics approval | 24 | This clinical trial has been approved by the Kilimanjaro Christian Medical College Research Ethics and Review Committee (CRERC), and the Tanzanian National Institute for Medical Research (NIMR) and registered to Pan African Clinical Trials Registry (PACTR).  Page 10 and 11 |
| Protocol amendments | 25 | Although there are no plans for any protocol modifications but in case it happens, they will be communicated to the all the responsible authorities beforehand.  Page 10 |
| Consent or assent | 26a | Persons legally responsible for the participants will be given thorough study details and clarifications and signed a free informed consent in front of Principle investigator.  Page 10 |
|  | 26b | Obtaining about 2mls of venous blood for complete blood count as a pre-operative routine will be explained and consented in the same study consent form.  Page 10 |
| Confidentiality | 27 | Confidentiality will be observed by using patients initials and hospital registration number in all study documents and not to be accessible by any person not responsible in the study. Use of coded data will be ensured to ensure maximum confidentiality.  Page 10 and 11 |
| Declaration of interests | 28 | Principal investigators declares no financial or other competing interests for the overall trial and at each study site.  Page 12 |
| Access to data | 29 | All research data will be stored under lock and under the custody of the principal investigator and supervisor (s) to prevent any illicit access to the data. At the end of the study, raw data will be stored in one soft copy storage device and folders will be locked in a special cabin at ENT department doctors’ office for a specific period.  Page 10 |
| Ancillary and post-trial care | 30 | Not applicable |
| Dissemination policy | 31a | Plans for Utilization and Dissemination of Information to  Kilimanjaro Christian Medical University College (KCMUCo), Kilimanjaro Christian Medical Centre (KCMC) and Arusha Lutheran Medical Centre (ALMC) Libraries, KCMC-Ear,Nose and Throat department, ALMC pediatric surgery department, Ministry of Health, Community Development, Gender, Elderly and Children (MoHCDEC) and its agencies. Local and International conferences, Manuscript for publication in the peer review Journal as well as policy brief note will be prepared.  Page 12 |
|  | 31b | Authorship will remain as the property of the research team as declared from the start of the study.  Page 12 |
|  | 31c | Full study protocol will be made available to public for unlimited access. |
| Appendices |  |  |
| Informed consent materials | 32 | Model consent form and other related documentation given to participants and authorised surrogates  English Informed Consent Form  **Kilimanjaro Christian Medical University College**  **Directorate of Postgraduate Studies**  **Department of Ear, Nose and Throat (ENT)**  **Informed Consent**  **[Informed Consent Form for _______________________________________________**  This informed consent form is for the parents/care takers of children from the ages of 2 years to 14 who are attending ENT Department being admitted for Tonsillectomy with or without adenoidectomy and who we are asking to participate in a research titled  **“Prophylactic Role Of Amoxicillin On Postoperative Morbidity After (Adeno)Tonsillectomy Among Children In Northern Tanzania: A Double Blind Randomized Placebo Controlled Trial.”**  **Dr. Denis Robert Katundu [Principal Investigator]**  **[KCMUCollege]**  **This Informed Consent Form has two parts:**  **1.Information Sheet (to share information about the study with you)**  **2.Certificate of Consent (for signatures if you agree that your child may participate)**  **You will be given a copy of the full Informed Consent Form**  **PART I: Information Sheet**  **Introduction**  I am Dr. Denis Robert Katundu, a 3^rd^ year resident doctor in ENT department and also working for the KCMC hospital. Together with my other colleagues we are doing research on the role of Amoxicillin on Tonsillectomy with or Without Adenoidectomy Morbidities in Northern Tanzania, prescription of amoxicillin post adenotonsillectomy is a which is very common practice in this hospital and country as well.  I am going to give you information and invite you to have your child participate in this research. You do not have to decide now whether or not you agree that your child may participate in the research. Before you decide, you can talk to anyone you feel comfortable with.  There may be some words that you do not understand. Please ask me to stop as we go through the information and I will take time to explain. If you have questions later, you can ask the study doctor or the staff.  **Purpose**  Drugs that kill or stop bacteria from harming human being (Antimicrobial agents) are commonly used in our hospital and community as well. They have been and still are used for many purposes including post surgery. In other parts of the world, there is a lot of current evidences for misuse of this drugs which have contributed to rapid growth of resistance of bacteria against these drugs. Resistant bacteria can not be treated once they cause disease. It has been found that Amoxicillin has no any preventive role to post tonsillectomy/adenotonsillectomy surgery in most of clinical researches which have been done in different parts of the world. Nevertheless amoxicillin is widely used in Tanzania in (adeno)tonsillectomy patients.. Purpose of this research is to look for any role of amoxicilline on post tonsillectomy/adenotonsillectomy morbidity in our setting.  **Type of Research Intervention**  This is a double blind randomized placebo controlled trial where Children will be randomly divided in two groups, one group will receive amoxicillin while the other group will receive a placebo . All will be closely followed-up.  **Participant selection**  Tonsillectomy with or without adenoidectomy is the major childhood surgery globally and especially in developing communities like Tanzania. Unnecessary exposure to antimicrobial agents is a major concern in children as due to the fact that parents/caretakers or healthcare providers are the one deciding for them. For children having a good indication of antimicrobial agents use and understanding which tonsillar microorganisms are currently resistance and sensitive to amoxicillin will help to ideally treat and minimize exposure of this drugs in this population.  We are inviting you to take part in this research because your child is living in this country where we still misuse many antibiotics especially amoxicillin due to little knowledge of why we he/she should receive use this drug post tonsillectomy/adenotonsillectomy and if there is any protection against post surgery morbidity. Your child doesn’t have any pre-surgical morbidity that’s a reason we are asking for your permission to allow your child to participate.  **Voluntary Participation**  Your decision to have your child participate in this study is entirely voluntary. It is your choice whether to have your child participate or not. If you choose not to consent, all the services you and your child receive in this department will continue and nothing will change. You may also choose to change your mind later and stop participating, even if you agreed earlier, and the services you and/or your child receives at the department will continue.  Prescribing 5 to 7 days of amoxicillin has been done in many setting before but nothing has been done in our country and Africa. In several studies, amoxicillin has notshown any prophylactic role on post tonsillectomy/adenotonsillectomy morbidity compared to not giving the antibiotic or giving a placebo. Not giving amoxicillin is of advantage as the child will be out of drug side effects such as rash, abdominal discomfort, diarrhoea, nausea, vomiting and worse hypersensitivity reaction..  Amoxicillin suspension we will be using is made by India based Company Sparsh Bio Tech Private Limited (branded as Spamox Oral Suspension of 125mg/5ml and 250mg/5ml concentration) while capsules of 250mg produce of North china pharmaceutical group corporation, these drugs have been approved in Tanzania by responsible authority, at KCMC hospital we will use any available amoxicillin regardless of the producers as long as its approved and available in the hospital pharmacy. It is called a antibiotic type of drug because it is used to treat a wide variety of bacterial infections its side effects include but not limited to allergic reaction, nausea, vomiting, or diarrhoea. The placebo will have no any known side effect.  **Procedures and Protocol**  Because in our setting we don’t know if the not giving amoxicillin is better than giving it for post tonsillectomy/adenotonsillectomy children in controlling the morbidity, we need to make comparisons. Children taking part in this research will be put into two groups which are selected by chance, as if by tossing a coin.  One group will get the placebo which is an inactive medicine looks like real medicine but it is not. It is a dummy or pretend medicine. It has no effect on a person because it has no real medicine in it. Sometimes when we want to know whether a new medicine is good, we give some people the new medicine and some people the pretend or dummy medicine. For the research to be good, it is important that you and your child do not know whether the real medicine or the pretend or dummy medicine was given. This is one of the best ways we have for knowing what the medicine we are testing really does.  The other group will get the amoxicillin which is currently used in this hospital and many places in Tanzania for post tonsillectomy/adenotonsillectomy children. It is important that neither you nor we know which of the two compounds your child will be given. This information will be in our files, but we will not look at these files until after the research is finished. This is the best way we have for testing the true effect without being influenced by what we think or hope might happen. We will then compare which of the two has the best results.  The healthcare workers will be looking after you and the other participants very carefully during the study. If we are concerned about what the medicines or treatment is doing, we will find out which medication your child is getting and make changes if necessary  If we find that the medicine that is being used does not have the desired effect, or not to the extent that we wish it to have, we will use what is called a “rescue medicine.”  **Description of the Process**  You may stay with your child during each of the visits and during some procedures on follow-ups.  In the next visit, your child will be taken to theatre for tonsillectomy/adenotonsillectomy during and after the procedure swab will be taken from the surface and deep of the tonsils and submitted to the lab. After surgery apart from antipains your child will be on post operative prophylaxis medication which neither you nor we will know, until later in the study, which vaccine your child was given. The medication will be given orally by a trained nurse. Same day after surgery, we will teach you how to provide post operative care in the hospital and at home also we will explain and show you how to handle the home based questionnaire.  We will ask you or person caring your child to give us the details of your child's health and illness related information through the same questionnaire. If you do not wish us to do that, please let us know. However, because your child's health records are very important for the study, if we cannot look at the health records, we will not be able to include your child in the study.  At the end of the study, during the last clinic follow-up we will tell you which of the two vaccines? your child was given.  Your child will receive the treatment for his/her condition according to hospital and national guidelines.  **Duration**  The research takes place for about 12 months in total but your child will only be followed for 14 days. During that time, it will be necessary for you to come to the clinic 3 days, for 10minutes each visit. Altogether, we will see you and your child 3 times post operatively.  **Side Effects**  The antibiotic given after surgerycan have some unwanted effects or some effects that we are not currently aware of. However, we will follow your child closely and keep track of these unwanted effects or any problems. We will give you a telephone number to call if you notice anything out of the ordinary, or if you have concerns or questions. You can also bring your child to this our ENT Clinic at anytime and ask to see [Doctor Denis R. Katundu, 0713828862].  We may use some other medicines to decrease the symptoms of the side effects or reactions. Or we may stop the use of one or more drugs. If this is necessary we will discuss it together with you and you will always be consulted before we move to the next step.  **Risks**  By participating in this research it is possible that your child will be at risk than he/she would otherwise be. There is a possibility that hypersensitivity to amoxicillin may happen as a result of taking this drug. While the possibility of this happening is very low, you should still be aware of the possibility. If something unexpected happens and harm does occur, we will provide your child with specialist care and the research grant will pay for it.  **Discomforts**  By participating in this research it is possible that your child may experience some discomfort such as the discomfort of the injections and surgical site swabs. There may be a slight hardening and/or swelling where the needle stick goes into the skin. This should disappear in one day. Your child may also be fussier than usual or more tired. These behaviors usually stop within one day but if you are concerned, please call me or come to the clinic.  **Benefits**  If your child participates in this research, he/she will have the following benefits: any interim illnesses will be treated at no charge to you. If your child falls sick during this period he/she will be treated free of charge. There may not be any other benefit for your child but his/her participation is likely to help us find the answer to the research question. There may not be any benefit to the society at this stage of the research, but future generations are sure to benefit.  **Reimbursements**  You will not be provided any incentive to take part in this research. However, you will be given a free thermometer and reimbursed with 5000 Tsh for your lost time and travel expense for each follow-up visit.  **Confidentiality**  The information that we collect from this research project will be kept confidential. Information about your child that will be collected from the research will be put away and no-one but the researchers will be able to see it. Any information about your child will have a number on it instead of his/her name. Only the researchers will know what his/her number is and we will lock that information up with a lock and key. It will not be shared with or given to anyone except [Dr Peter S Shija, a research supervisor]  **Sharing of the results**  The knowledge that we get from this study will be shared with you before it is made widely available to the public. Confidential information will not be shared. There will be small meetings in the community and these will be announced. Afterwards, we will publish the results in order that other interested people may learn from our research.  **Right to Refuse or Withdraw**  You do not have to agree to your child taking part in this research if you do not wish to do so and refusing to allow your child to participate will not affect your treatment or your child's treatment at this Centre in any way. You and your child will still have all the benefits that you would otherwise have at KCMC Hospital. You may stop your child from participating in the research at any time that you wish without either you or your child losing any of your rights as a patient here. Neither your treatment nor your child's treatment at this Centre will be affected in any way.  **Alternatives to participating**  If you do not wish your child to take part in the research, your child will be provided with the established standard treatment available at the ENT department of KCMC hospital. All people undergoing adenotonsillectomy/tonsillectomy will be subjected to amoxicillin for 5 days.  **Who to Contact**  If you have any questions you may ask them now or later, even after the study has started. If you wish to ask questions later, you may contact any of the following:  [Dr Denis R Katundu, 3010Moshi, 0713828862/katundu101@gmail.com]  [Dr Peter S Shija 3010Moshi, 0754392916 / [shijapsn@yahoo.com](mailto:shijapsn@yahoo.com)]  [National Health Research Ethics Sub-Committee (NatHREC), National Institute for Medical Research (NIMR), P.O. Box 9653 Dar es Salaam, +255 22 212 14 00]  This proposal has been reviewed and approved by KMCUCollege-Clinical Research Ethical Committee, NIMR and registered by TFDA through Tanzania Clinical Trials Registry, which is a committee whose task it is to make sure that research participants are protected from harm. If you wish to find about more about the IRB, contact [Dr. Mramba Nyindo]  **PART II: Certificate of Consent**  **Certificate of Consent**  I have been invited to have my child participate in research of amoxicillin prophylactic role in post tonsillectomy/adenotonsillectomy morbidity.  **I have read the foregoing information, or it has been read to me. I have had the opportunity to ask questions about it and any questions that I have asked have been answered to my satisfaction. I consent voluntarily for my child to participate as a participant in this study.**  **Name of Participant__________________**  **Name of Parent or Guardian_______________**  **Signature of Parent or Guardian ___________________**  **Date ___________________________**  **Day/month/year**  **If illiterate**  **I have witnessed the accurate reading of the consent form to the parent of the potential participant, and the individual has had the opportunity to ask questions. I confirm that the individual has given consent freely.**  ***Name of witness_____________________ AND Thumb print of parent***  ***Signature of witness ______________________***  **Date ________________________**    **Statement by the researcher/person taking consent**  **I have accurately read out the information sheet to the parent of the potential participant, and to the best of my ability made sure that the person understands that the following will be done:**  **1. Blood will be taken for Full blood picture, liver and renal function tests**  **2. Surface and tonsillar swab for microbiology and microbial susceptibility analysis**  **3. Followed up at ENT Clinic together with filling a home based questionnaire.**  **I confirm that the parent was given an opportunity to ask questions about the study, and all the questions asked by the parent have been answered correctly and to the best of my ability. I confirm that the individual has not been coerced into giving consent, and the consent has been given freely and voluntarily.**    **A copy of this Informed Consent Form has been provided to the participant.**  **Name of Researcher/person taking the consent______________________**  **Signature of Researcher /person taking the consent__________________**  **Date __________________________** |
| Biological specimens | 33 | Followed by insertion of a Boyle-Davies mouth gag, transorally under direct vision by using a sterilized cotton-tipped applicators surface tonsillar swabs will be taken on both sides and secured one in Copan Liquid Amies Elution Swab (ESwab) Collection and Transport System and Universal Transport Media (UTM) separately.  Routine dissection tonsillectomy will be performed, haemostasis will be secured with monopolar coagulation diathermy. For guillotine tonsillectomy, the Doyen mouth gag will be used to open the mouth widely and the tonsils will be removed using Poppers' haemostatic guillotine. During electrodissection of palatine tonsils, Valleylab^®^ Force 1B with monopolar and bipolar outputs will be used for electrocautery with standardized 20-Watts monopolar technique.  All dissected tonsils will be deeped in Povidone for about 30 seconds and one tonsil will be choosen, rinsed out using sterilized physiologic normal saline. Afterwards, using a sterile surgical blade the tonsils will be divided into two parts and the samples will be taken from the core of the tonsils by sterilized swabs. These swabs will be secured in Copan Liquid Amies Elution Swab (ESwab) Collection and Transport System and UTM separatelly. Then, for patients operated at KCMC both swabs will be dispatched within 30 minutes to the microbiology section of research laboratory (KCRI) but samples from ALMC will be stored and transported same day to KCMC research laboratory maintaining temperature between 2-8°C (fig 02).  One surface and one core swabs will secured in Copan Liquid Amies Elution Swab (ESwab) and one surface and one core swabs in UTM swab separately. All ESwabs will be transported immediately to microbiology lab while UTM swabs will be stored at -80 celcius for possible further PCR and serotyping analysis.  ESwab Specimens will be incubated at 37°C for 24 h. After 24 hours of incubation, subcultures on blood agar from growing microorganism will be prepared. The isolated bacteria will then be gram stained and microscopically investigated. Antibiotic sensitivity tests will be carried out for pathogenic isolates by disc diffusion technique respective of tonsillar surface or core specimen.  Antibiotic discs for the commonly used antibiotics will be used, comprises of ampicillin, amoxycillin, amoxacillin-clavulanic acid, erythromycin, azithromycin ceftriaxon, cefpodoxim, cefixime. Reading of the plates will be done according to globally accepted and standardized microbiological procedures.  Patients will be followed-up serially at the clinic and through regular cellphones on 07th and 14th days post operatively at ENT by the researcher where wound inspection will be done and questionnaire information will be captured and the third swab being taken on 14^th^ day to asses for recolonization around the surgical site. Patients fare to study site, consultation fee, axillary thermometers and any complication related to the study will be taken care by the study during follow-up period. |

|  | **January 2019-March 2020** | | | | | | | | | |
| --- | --- | --- | --- | --- | --- | --- | --- | --- | --- | --- |
|  | **Clinic visit** | **Admission** | **Post-(Adeno)tonsillectomy (days)** | | | | | | | |
| **TIMEPOINT** | **Registry** | **Scheduled** | **0** | **1** | **2** | **3** | **4** | **5** | ***7*** | **14** |
| **ENROLMENT** |  |  |  |  |  |  |  |  |  |  |
| **Eligibility screen** | X |  |  |  |  |  |  |  |  |  |
| **Informed consent** |  | X |  |  |  |  |  |  |  |  |
| **Capturing Baseline information** |  | X |  |  |  |  |  |  |  |  |
| **Preoperative review and qualifying the inclusion criteria** |  | X |  |  |  |  |  |  |  |  |
| **participant details delivered to pharmacist for randomization** |  | X |  |  |  |  |  |  |  |  |
| **Blinded research Nurse obtain randomized interventions** |  |  | X |  |  |  |  |  |  |  |
| **INTERVENTIONS:** |  |  |  |  |  |  |  |  |  |  |
| ***[Amoxicillin group]*** |  |  | ♦ |  |  |  |  | ♦ |  |  |
| ***[Placebo group]*** |  |  | ♦ |  |  |  |  | ♦ |  |  |
| **ASSESSMENTS:** |  |  |  |  |  |  |  |  |  |  |
| **Baseline Valiables**  Gender, age group (2-4 years, 5-8 years and 9-14 years), residence (rural or urban) and research centre (KCMC or ALMC***)*** | X | X |  |  |  |  |  |  |  |  |
| ***Primary outcomes***   1. Hemorrhage 2. Raised Temperature 3. Pain |  |  |  | X | X | X | X | X | X |  |
| ***Secondary outcomes***   1. Time until normal diet is resumed 2. Time until normal activities are resumed 3. Adverse events such as a rash, vomiting, diarrhea and anaphylaxis. 4. Microbial recolonization of the tonsillar beds |  |  |  | X | X | X | X | X | X |  |
| ***Post operative***   1. tonsillar niche swab 2. wound assessment |  |  |  |  |  |  |  |  |  | X |
| ***Participation signoff*** |  |  |  |  |  |  |  |  |  | X |

Figure01. Trial schedule for enrolment, interventions, and follow-up/assessments

**References**

1. Ingram, D.G. and N.R. Friedman, *Toward Adenotonsillectomy in Children: A Review for the General Pediatrician.* JAMA Pediatr, 2015. **169**(12): p. 1155-61.

2. Oburra, H.O. and M. Idenya, *Frequency of adenotonsillectomy in some Nairobi hospitals.* East Afr Med J, 2001. **78**(7): p. 338-42.

3. Rohlfing, M.L., et al., *Investigation of Postoperative Oral Fluid Intake as a Predictor of Postoperative Emergency Department Visits After Pediatric TonsillectomyOral Fluid Intake and Adverse Outcomes After Pediatric TonsillectomyOral Fluid Intake and Adverse Outcomes After Pediatric Tonsillectomy.* JAMA Otolaryngology–Head & Neck Surgery, 2016. **142**(4): p. 357-363.

4. Egeli, E., et al., *Can post-adenotonsillectomy morbidity be reduced by intravenous 24 h hydration in pediatric patients following adenotonsillectomy?* Int J Pediatr Otorhinolaryngol, 2004. **68**(8): p. 1047-51.

5. Dhiwakar, M., et al., *Antibiotics to reduce post-tonsillectomy morbidity.* Cochrane Database Syst Rev, 2012. **12**: p. Cd005607.

6. Horumpende, P.G., et al., *Prescription and non-prescription antibiotic dispensing practices in part I and part II pharmacies in Moshi Municipality, Kilimanjaro Region in Tanzania: A simulated clients approach.* PLOS ONE, 2018. **13**(11): p. e0207465.

7. Pynnonen, M., et al., *Coblation versus other surgical techniques for tonsillectomy.* Cochrane Database of Systematic Reviews, 2017(8).

8. Pinder, D.K., H. Wilson, and M.P. Hilton, *Dissection versus diathermy for tonsillectomy.* Cochrane Database of Systematic Reviews, 2011(3).

9. Bauer, A.W., et al., *Antibiotic Susceptibility Testing by a Standardized Single Disk Method.* American Journal of Clinical Pathology, 1966. **45**(4_ts): p. 493-496.

------------------------------------------------------------------------------------------------------------------------

*It is strongly recommended that this checklist be read in conjunction with the SPIRIT 2013 Explanation & Elaboration for important clarification on the items. Amendments to the protocol should be tracked and dated. The SPIRIT checklist is copyrighted by the SPIRIT Group under the Creative Commons “[Attribution-NonCommercial-NoDerivs 3.0 Unported](http://www.creativecommons.org/licenses/by-nc-nd/3.0/)” license.
